# Legumain Promotes Atherosclerotic Vascular Remodeling

**DOI:** 10.3390/ijms20092195

**Published:** 2019-05-04

**Authors:** Nana Ozawa, Yuki Sato, Yukari Mori, Hiroko Masuda, Mao Yamane, Yuka Yamamoto, Remina Shirai, Rena Watanabe, Kengo Sato, Yusaku Mori, Tsutomu Hirano, Takuya Watanabe

**Affiliations:** 1 Laboratory of Cardiovascular Medicine, Tokyo University of Pharmacy and Life Sciences, Tokyo 192-0392, Japan; s139021@toyaku.ac.jp (N.O.); s139052@toyaku.ac.jp (Y.S.); s147067@toyaku.ac.jp (Y.M.); s159095@toyaku.ac.jp (H.M.); s159111@toyaku.ac.jp (M.Y.); s159112@toyaku.ac.jp (Y.Y.); rshirai@kumamoto-u.ac.jp (R.S.); rena.watanabe@rd.witc.co.jp (R.W.); ksato@toyaku.ac.jp (K.S.); 2 Division of Diabetes, Metabolism, and Endocrinology, Department of Medicine, Showa University School of Medicine, Tokyo 142-8555, Japan; torigoe1234@yahoo.co.jp (Y.M.); hirano@med.showa-u.ac.jp (T.H.)

**Keywords:** legumain, atherosclerosis, neointimal thickness, remodeling, macrophage, VSMC, ECM

## Abstract

Legumain, a recently discovered cysteine protease, is increased in both carotid plaques and plasma of patients with carotid atherosclerosis. Legumain increases the migration of human monocytes and human umbilical vein endothelial cells (HUVECs). However, the causal relationship between legumain and atherosclerosis formation is not clear. We assessed the expression of legumain in aortic atheromatous plaques and after wire-injury-induced femoral artery neointimal thickening and investigated the effect of chronic legumain infusion on atherogenesis in *Apoe*^−/−^ mice. We also investigated the associated cellular and molecular mechanisms in vitro, by assessing the effects of legumain on inflammatory responses in HUVECs and THP-1 monocyte-derived macrophages; macrophage foam cell formation; and migration, proliferation, and extracellular matrix protein expression in human aortic smooth muscle cells (HASMCs). Legumain was expressed at high levels in atheromatous plaques and wire injury-induced neointimal lesions in *Apoe*^−/−^ mice. Legumain was also expressed abundantly in THP-1 monocytes, THP-1 monocyte-derived macrophages, HASMCs, and HUVECs. Legumain suppressed lipopolysaccharide-induced mRNA expression of vascular cell adhesion molecule-1 (*VCAM1*), but potentiated the expression of interleukin-6 (*IL6*) and E-selectin (*SELE*) in HUVECs. Legumain enhanced the inflammatory M1 phenotype and oxidized low-density lipoprotein-induced foam cell formation in macrophages. Legumain did not alter the proliferation or apoptosis of HASMCs, but it increased their migration. Moreover, legumain increased the expression of collagen-3, fibronectin, and elastin, but not collagen-1, in HASMCs. Chronic infusion of legumain into *Apoe*^−/−^ mice potentiated the development of atherosclerotic lesions, accompanied by vascular remodeling, an increase in the number of macrophages and ASMCs, and increased collagen-3 expression in plaques. Our study provides the first evidence that legumain contributes to the induction of atherosclerotic vascular remodeling.

## 1. Introduction

Atherosclerosis is characterized by a complex process of vascular injury; inflammation, with monocyte adhesion to endothelial cells (ECs); lipid deposition within macrophage foam cells; neointimal hyperplasia, involving vascular smooth muscle cells (VSMCs); and extracellular matrix (ECM) remodeling [1,2]. Vascular inflammation is characterized by the up-regulation of tumor necrosis factor-α (TNF-α), interleukin (IL)-6, monocyte chemoattractant protein-1 (MCP-1), pentraxin-3, intercellular adhesion molecule-1 (ICAM-1), vascular cell adhesion molecule-1 (VCAM-1), and E-selectin in ECs and a pro-inflammatory M1 phenotype in macrophages [3,4,5]. Macrophage foam cell formation is characterized by cholesterol ester accumulation, which depends on the balance between the uptake of oxidized low-density lipoprotein (oxLDL) through scavenger receptor class A (SR-A) or CD36 and the efflux of free cholesterol, controlled by ATP-binding cassette transporter A1 (ABCA1) [6,7]. Intracellular cholesterol ester accumulation is also modulated by the balance between cholesterol esterification by acyl-coenzyme A:cholesterol acyltransferase-1 (ACAT-1) and hydrolysis by neutral cholesterol ester hydrolase (NCEH) [6,7]. VSMCs contribute to the development of atherosclerosis and restenosis after angioplasty by their migration, proliferation, and production of ECM components, such as collagens, elastin, fibronectin, and matrix metalloproteinases (MMPs) [8,9,10]. 

Legumain (433 amino acids), also known as asparagine endopeptidase, belongs to the C13 family of cysteine proteases [11,12]. Human and mouse legumain amino acid sequences are 83% identical [13]. Legumain plays a role in the processing of antigens for MHC class II presentation in the lysosomes of antigen presenting cells [14] and it supports human Th1 cell induction [15]. Legumain is abundantly expressed in testes, kidneys, bone marrow stromal cells, monocytes, macrophages (M1 and M2), dendritic cells, and adipocytes [16,17,18,19,20,21,22,23]. The expression and secretion of legumain increase during differentiation from monocytes to macrophages [17,21]. Legumain is up-regulated by hypoxia, oxLDL, macrophage colony-stimulating factor, and IL-4 but not by lipopolysaccharide (LPS) [17,23,24,25]. Legumain increases the migration of human monocytes [26], but inhibits the oxLDL-induced apoptosis of human macrophages [25]. Legumain increases the migration and proliferation of human umbilical vein endothelial cells (HUVECs) [26]. Recent clinical studies have shown that legumain is expressed in carotid plaques and is present at a high concentration in the plasma of patients with carotid atherosclerosis [22,27,28,29]. However, the specific role of legumain in the pathophysiology of atherosclerosis has not yet been determined.

In the present study, we assessed the expression of legumain in atheromatous plaques and wire-injury-induced neointimal lesions. Moreover, we studied the effects of chronic legumain infusion on the development of atherosclerosis in apolipoprotein E-deficient (*Apoe*^−/−^) mice, an established animal model of atherosclerosis. The cellular and molecular mechanisms mediating the effects of legumain were examined in vitro, by assessing inflammatory responses in HUVECs; the inflammatory phenotype and foam cell formation in human THP-1 monocyte-derived macrophages; and migration, proliferation, and ECM production in human aortic smooth muscle cells (HASMCs) after legumain treatment. 

## 2. Results

### 2.1. Expression of Legumain in Mouse Atherosclerotic Lesions

**Figure 1:**
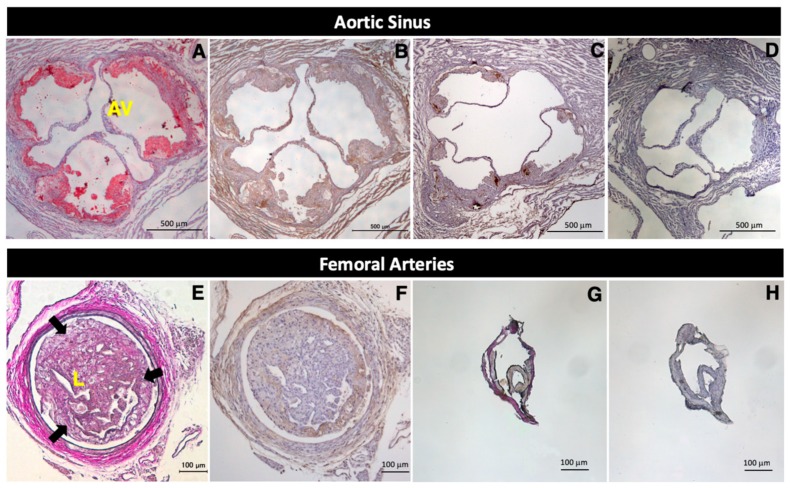
Expression of legumain in atheromatous plaques and neointimal lesions following wire injury in *Apoe*^−/−^ mice. *Apoe*^−/−^ mice were fed a high-cholesterol diet from the age of 13 weeks. Tissues were stained with Oil Red O (**A**), an anti-legumain antibody (**B**–**D**,**F**,**H**), and Elastica-Van Gieson (**E**,**G**). Nuclei were stained with hematoxylin. Atheromatous plaques (reddish areas stained by Oil Red O) were observed in the aortas of 21-week-old *Apoe*^−/−^ mice (**A**). Marked neointimal hyperplasia (reddish-purple areas surrounded by arrows) was observed in the obstructed left femoral artery following wire injury in 15-week-old *Apoe*^−/−^ mice (**E**). In *Apoe*^−/−^ mice, legumain (dark blown) was expressed at high levels in the advanced and early-stages of aortic atheromatous plaques at 21 weeks old (**B**) and 17 weeks old (**C**), respectively, and in left femoral artery neointimal lesions after wire injury at 15 weeks old (**F**). Legumain expression was not observed in the non-atherosclerotic aortas at 13 weeks old (**D**) or in the right normal femoral arteries at 15 weeks old (**H**). Each experiment was performed on at least two independent occasions to ensure reproducibility. AV = aortic valve, L = lumen.

Marked atheromatous plaques were observed in the aortas of 21-week-old *Apoe*^−/−^ mice (Figure 1A). Legumain was abundantly expressed in advanced and early-stage atheromatous plaques in the aortas of 21-week-old and 17-week-old *Apoe*^−/−^ mice, respectively (Figure 1B,C), but was not detected in the non-atherosclerotic aortas of 13-week-old control *Apoe*^−/−^ mice (Figure 1D).

Neointimal hyperplasia was observed in the obstructed left femoral arteries following wire injury in 15-week-old *Apoe*^−/−^ mice (Figure 1E). Abundant legumain expression was also detected in marked neointimal lesions in the injured left femoral arteries (Figure 1F). In contrast, in the non-injured right femoral arteries (Figure 1G), legumain was not expressed in the normal right femoral arteries of 15-week-old *Apoe*^−/−^ mice (Figure 1H).

### 2.2. Effects of Legumain on Atherosclerotic Lesion Development in Apoe^−/−^ Mice

**Figure 2:**
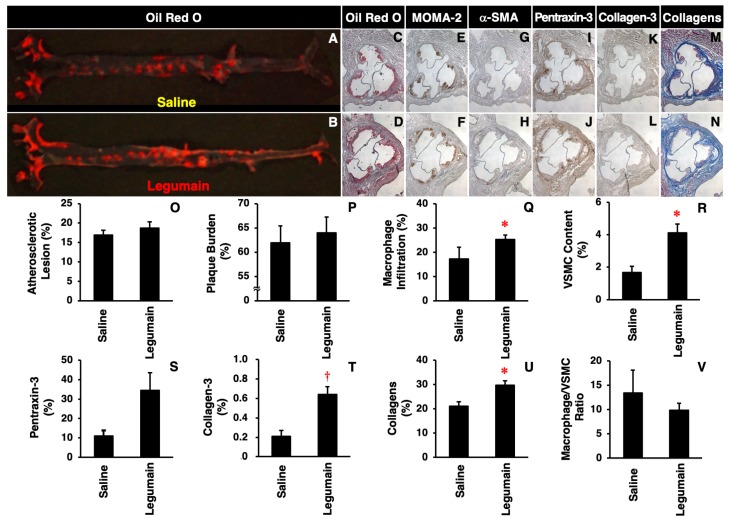
Effects of legumain on atherosclerotic lesion development in *Apoe*^−/−^ mice. Mice were sacrificed after a four-week infusion of legumain (5 μg/kg/h, *n* = 8) or saline (control, *n* = 4). (**A**,**B**) Atherosclerotic lesions on the aortic luminal surface were stained with Oil Red O. (**C**–**N**) Cross-sections of the aortic sinus wall were stained with Oil Red O, anti-MOMA-2 (monocytes/macrophages), anti-α-SMA (vascular smooth muscle cell, VSMCs), anti-pentraxin-3 (vascular inflammation), anti-collagen-3 antibodies, or Masson’s Trichrome (collagen fibers). Nuclei were stained with hematoxylin. (**O**–**U**) The degree of staining was compared between legumain- and saline-treated groups. (**V**) The macrophage/VSMC ratio was calculated as a marker of plaque instability. Data are expressed as means ± SEM. * *p* < 0.05, ^†^ *p* < 0.01 vs. saline. Analyzed by unpaired Student’s *t* test.

There were no significant differences in body weight; food intake; systolic or diastolic blood pressure; or fasting plasma levels of glucose, total cholesterol, triglycerides, free fatty acids, or insulin between *Apoe*^−/−^ mice treated with saline and those treated with legumain (Table 1). However, chronic legumain infusion tended to aggravate atherosclerotic lesions on the aortic luminal surface (Figure 2A,B,O), with somewhat increased plaque burden on the aortic wall and vascular inflammation (pentraxin-3 expression) (Figure 2C,D,I,J,P,S); there were no significant differences. Legumain significantly increased the number of macrophages, VSMCs, and collagen fibers as well as collagen-3 expression in the aortic sinus wall (Figure 2E–H,K–N,Q,R,T,U). Legumain tended to reduce the macrophage/VSMC ratio, which is a surrogate marker of atheromatous plaque instability (Figure 2V).

### 2.3. Expression of Legumain in Human Vascular Cells

**Figure 3:**
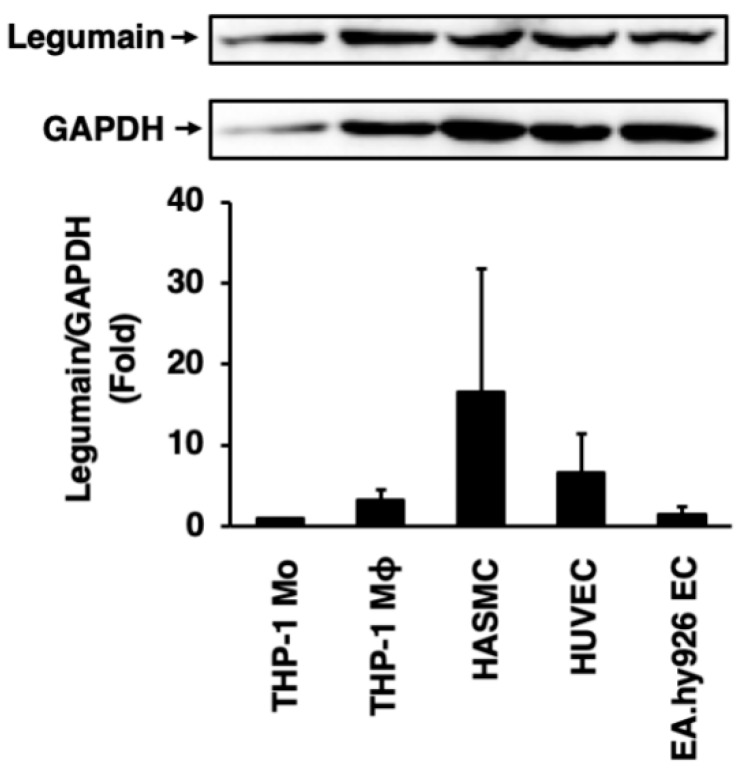
Expression of legumain in human vascular cells. Legumain expression in THP-1 monocytes (Mo), THP-1 monocyte-derived macrophages (Mϕ), human aortic smooth muscle cells (HASMCs), human umbilical vein endothelial cells (HUVECs), and EA.hy926 endothelial cells (ECs) were assessed by immunoblotting. Glyceraldehyde-3-dehydrogenase (GAPDH) served as a loading control. Top: Representative immunoblots of legumain and GAPDH. Bottom: Densitometric data, after normalization to GAPDH, from three independent experiments (*n* = 3).

Legumain was abundantly expressed in THP-1 monocytes, THP-1 monocyte-derived macrophages, HASMCs, HUVECs, and EA.hy926 ECs (Figure 3). Legumain expression levels were higher in macrophages than in monocytes, but were highest in HASMCs, among all vascular cells tested (Figure 3). 

### 2.4. Effects of Legumain on Inflammatory Responses in HUVECs

**Figure 4:**
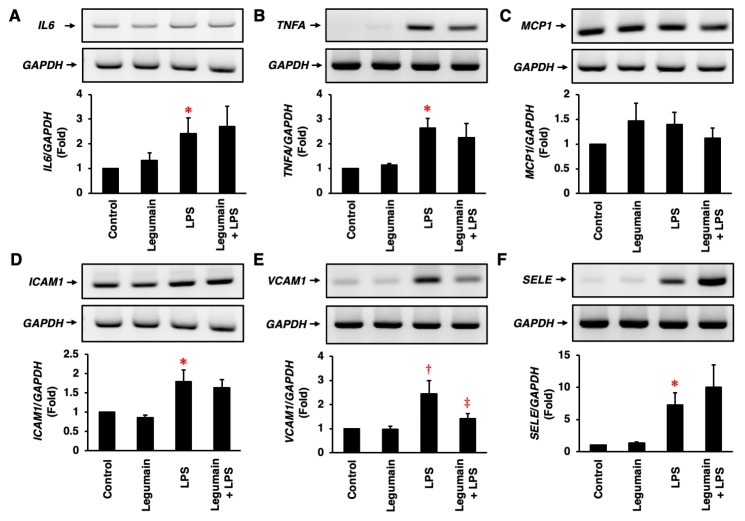
Effects of legumain on inflammatory responses in human ECs. The effects of legumain (25 ng/mL) on the lipopolysaccharide (LPS) (1 μg/mL)-induced mRNA expression of *IL6* (**A**), *TNFA* (**B**), *MCP1* (**C**), *ICAM1* (**D**), *VCAM1* (**E**), and *SELE* (**F**) in HUVECs were assessed by RT-PCR. Four independent single-assays were performed using four different cultures (*n* = 4). *GAPDH* served as a control. Top: Representative mRNA expression results. Bottom: Densitometric data after normalization to *GAPDH*. * *p* < 0.005, ^†^ *p* < 0.05 vs. control; ^‡^ *p* < 0.05 vs. LPS. Analyzed by one-way analysis of variance (ANOVA) with Bonferroni’s test.

Legumain had no significant effect on the mRNA expression of *IL6*, *TNFA*, *ICAM1*, *VCAM1*, or *SELE*, but LPS significantly stimulated the expression of these mRNAs in HUVECs (Figure 4A,B,D–F). However, legumain tended to increase *MCP1* mRNA expression to the same extent as LPS (Figure 4C). In HUVECs, legumain significantly suppressed the LPS-induced mRNA expression of *VCAM1* (Figure 4E) and tended to decrease LPS-induced *TNFA* and *MCP1* expression (Figure 4B,C), but it did not affect LPS-induced *ICAM1* expression (Figure 4D). In contrast, legumain tended to increase the LPS-induced mRNA expression of *IL6* and *SELE* in HUVECs (Figure 4A,F); there were no significant differences.

### 2.5. Effects of Legumain on the Inflammatory Phenotype of Human Macrophages

**Figure 5:**
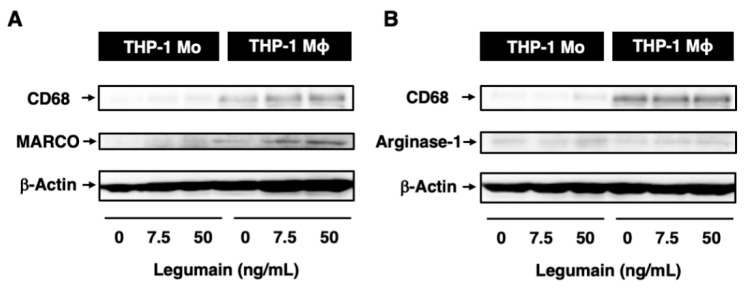
Effects of legumain on inflammatory phenotypes in human monocytes (Mo)/macrophages (Mϕ). The effects of legumain on the inflammatory phenotype of THP-1 monocyte-derived macrophages were assessed by immunoblotting. (**A**,**B**) CD68, MARCO, and arginase-1 were used as markers of macrophage differentiation, M1 macrophages, and M2 macrophages, respectively. β-Actin served as a loading control. A representative set of reproducible results are shown from five independent experiments.

After 6 days of culture, the differentiation of THP-1 monocytes to macrophages was confirmed by increased expression of CD68, a macrophage differentiation marker (Figure 5A,B). Legumain did not affect monocyte differentiation to macrophages. However, legumain (50 ng/mL) increased the expression of macrophage receptor with collagenous structure (MARCO), an M1 macrophage marker, but not arginase-1, an M2 macrophage marker, during differentiation (Figure 5A,B). 

### 2.6. Effects of Legumain on Foam Cell Formation

**Figure 6:**
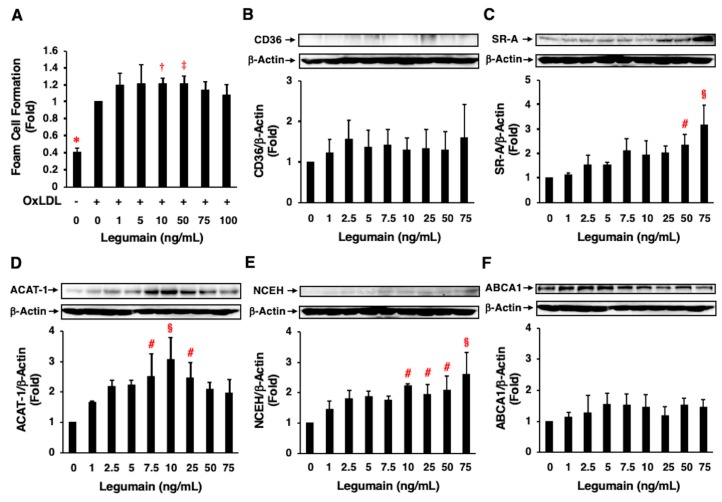
Effects of legumain on foam cell formation and relevant protein expression in human macrophages. (**A**–**F**) The effects of legumain on oxidized low-density lipoprotein (oxLDL)-induced foam cell formation and related protein expression in THP-1 monocyte-derived macrophages were assessed by cholesterol esterification assay and immunoblotting, respectively. (**A**) Seven independent experiments were performed (*n* = 7). 1-fold = 5.15 ± 0.67 nmol/mg total cellular protein. (**B**–**F**) Four independent experiments were performed (*n* = 4). Top: Representative immunoblots of CD36 (**B**), scavenger receptor class A (SR-A) (**C**), acyl-coenzyme A:cholesterol acyltransferase-1 (ACAT-1) (**D**), neutral cholesterol ester hydrolase (NCEH) (**E**), and ATP-binding cassette transporter A1(ABCA1) (**F**). β-Actin served as a loading control. Bottom: Densitometric data after normalization to β-actin. (**A**) * *p* < 0.0001, ^†^ *p* < 0.01, ^‡^ *p* < 0.05 vs. 0 ng/mL of legumain with oxLDL (+), (**B**–**F**) ^#^ *p* < 0.05, ^§^ *p* < 0.005 vs. 0 ng/mL of legumain. Analyzed by one-way ANOVA with Bonferroni’s test.

Treatment of THP-1 monocyte-derived macrophages with OxLDL significantly increased foam cell formation (Figure 6A). Further, legumain (10, 50 ng/mL) significantly enhanced oxLDL-induced foam cell formation (Figure 6A). Legumain significantly increased the protein expression of SR-A, ACAT-1, and NCEH in a concentration-dependent manner (Figure 6C–E) but did not significantly alter the expression of CD36 or ABCA1 (Figure 6B,F). 

### 2.7. Effects of Legumain on Migration, Proliferation, and Apoptosis of HASMCs

**Figure 7:**
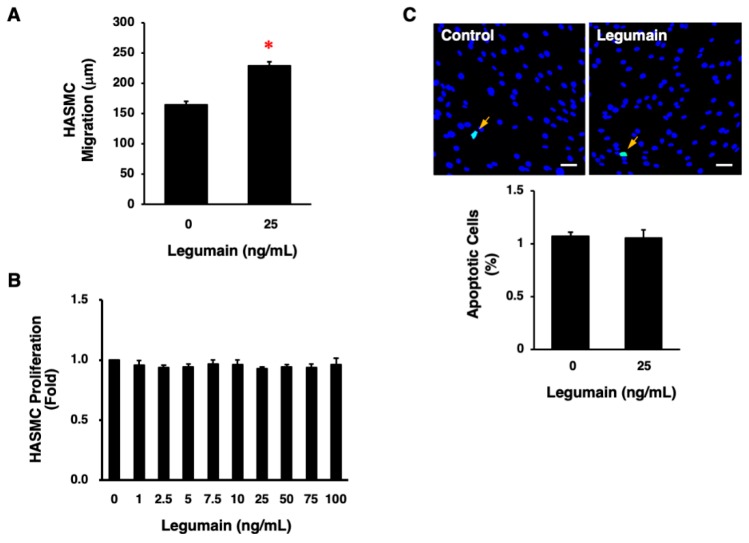
Effects of legumain on the migration, proliferation, and apoptosis of HASMCs. (**A**) The effect of legumain on HASMC migration was determined in 10 cells per well using a BIOREVO BZ-9000 microscope. Three independent experiments were performed (*n* = 30). * *p* < 0.0001. Analyzed by unpaired Student’s *t* test. (**B**) The effect of legumain on HASMC proliferation was determined by WST-8 assay. Three independent experiments were performed (*n* = 3). (**C**) The effect of legumain on HASMC apoptosis was evaluated by detecting apoptotic cells (green) using a terminal deoxynucleotidyl transferase-mediated deoxyuridine triphosphate-biotin nick end-labeling (TUNEL) assay. Nuclei were co-stained with 6-diamidino-2-phenylindole (blue). The graph indicates the percentage of apoptotic cells in three independent experiments (*n* = 3). Scale bar = 100 μm.

Legumain (25 ng/mL) significantly increased the migration of HASMCs (*p* < 0.0001, Figure 7A). However, legumain did not significantly affect the proliferation or apoptosis of HASMCs (Figure 7B,C). 

### 2.8. Effects of Legumain on ECM Protein Expression in HASMCs

**Figure 8:**
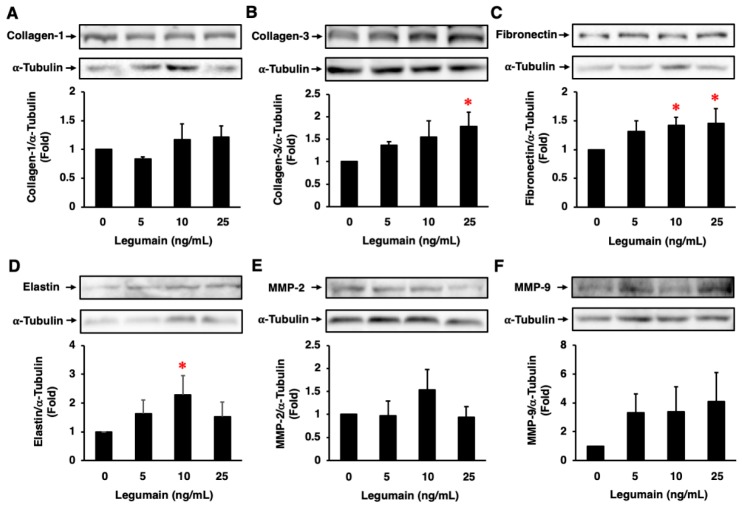
Effects of legumain on extracellular matrix (ECM) protein expression in HASMCs. The effects of legumain on collagen-1 (**A**), collagen-3 (**B**), fibronectin (**C**), elastin (**D**), MMP-2 (**E**), and MMP-9 (**F**) expression were assessed by immunoblotting (*n* = 4–7). α-Tubulin served as a loading control. Top: Representative immunoblots. Bottom: Densitometric data after normalization to α-tubulin. * *p* < 0.05 vs. 0 ng/mL of legumain. Analyzed by one-way ANOVA with Bonferroni’s test.

**Figure 9:**
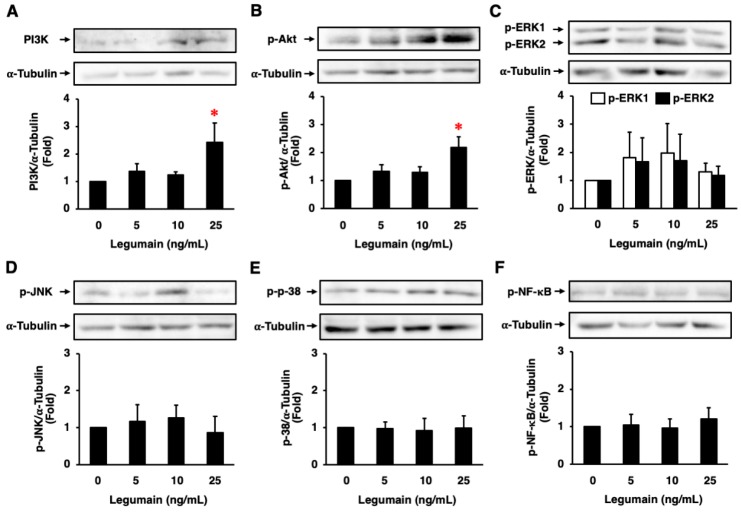
Effects of legumain on intracellular signal transduction in HASMCs. The protein expression/phosphorylation levels of PI3K (**A**), Akt (**B**), ERK1/2 (**C**), JNK (**D**), p38 (**E**), and NF-κB (**F**) were assessed by immunoblotting (*n* = 3–6). α-Tubulin served as a loading control. Top: Representative immunoblots. Bottom: Densitometric data after normalization to α-tubulin. * *p* < 0.05 vs. 0 ng/mL of legumain. Analyzed by one-way ANOVA with Bonferroni’s test.

Legumain did not significantly affect collagen-1 protein expression in HASMCs (Figure 8A). However, legumain significantly increased the protein expression of collagen-3, fibronectin, and elastin (Figure 8B–D), and tended to increase the protein expression of MMP-2 and MMP-9 in HASMCs (Figure 8E,F). Moreover, legumain significantly increased phosphoinositide 3-kinase (PI3K) expression and Akt phosphorylation (Figure 9A,B) and tended to increase extracellular signal-regulated kinase (ERK) 1/2 phosphorylation in HASMCs (Figure 9C). However, legumain did not significantly affect the phosphorylation of c-jun N-terminal kinase (JNK), p38, or nuclear factor-κB (NF-κB) (Figure 9D–F).

## 3. Discussion

This is the first demonstration that legumain, a newly discovered cysteine protease, potentiates atherosclerotic vascular remodeling. Legumain enhances macrophage foam cell formation and VSMC migration; increases collagen-3, fibronectin, and elastin expression in VSMCs in vitro; and induces the formation of atherosclerotic lesions, with increased numbers of macrophages and VSMCs and increased collagen-3 expression in *Apoe*^−/−^ mice in vivo. The effects of legumain on atherogenesis are not as strong as the effects of other vasoactive agents [30,31]. However, it is worth noting that legumain did promote atherosclerotic vascular remodeling, associated with increased ECM production via the PI3K/Akt pathway, in VSMCs. 

Previous studies have shown that legumain regulates ECM remodeling by inducing the degradation of collagen-1 and fibronectin [23,32] and exerts anti-fibrotic effects by attenuating the deposition of collagen and fibronectin in a mouse model of obstructive nephropathy [33]. A recent study has shown that legumain increases the synthesis of ECM proteins by activating MMP-2/transforming growth factor-β1 signaling in pulmonary artery VSMCs in a mouse model of pulmonary hypertension [24]. When considered in integration, legumain may contribute to tissue remodeling by repeating alternately the scrap and build of ECM under various pathophysiological conditions. In addition, a previous study has shown that the expression levels of legumain are higher in unstable plaques than in stable plaques in human carotid arteries [27]. Because legumain is co-expressed with MMP, it is regarded as a contributor to plaque rupture [27]. In our study, legumain increased the number of intraplaque collagen fibers and increased the expression of collagen-3, fibronectin, and elastin in VSMCs. Moreover, it decreased the macrophage/VSMC ratio, which is a marker of plaque instability. Our findings suggest that legumain may play a key role in the elasticity and stabilization of atheromatous plaques. Further studies are needed to determine whether legumain can stabilize plaques at the advanced atherosclerotic phase in *Apoe*^−/−^ mice. 

The trafficking of legumain in and outside the cell has been reported by Dall E and Brandstetter H [14,34]. Prolegumain is translocated via the endoplasmic reticulum and Golgi apparatus to the endo-lysosomal system, where it is activated to legumain as asparaginyl endopeptidase with acidic pH [34]. Endo-lysosomal pH is decreased by IL-6 and increased by IL-10, leading to the activation and inactivation of legumain, respectively [14]. Legumain may be extracellularly secreted directly via the Golgi apparatus or indirectly via the endosomal system. Extracellular legumain may re-enter the cell via endocytosis or directly via translocation to the cytoplasm [14]. However, the receptors for legumain have not been identified so far. These findings suggest that legumain may act in an autocrine/paracrine manner. Therefore, legumain is also regarded as a kind of local and systemic hormone.

Several studies have shown that plasma concentrations of legumain are ~0.6 and ~1.5 ng/mL in healthy subjects [22,24] and ~2.0 ng/mL in patients with carotid atherosclerosis [22]. The concentrations of legumain required to elicit THP-1 monocyte-derived macrophage, HUVEC, and HASMC responses in the present study were 7.5–75 ng/mL. Local levels of vasoactive agents, produced by vascular cells, may increase to similar concentrations and act in an autocrine/paracrine manner [35,36]. In addition, different concentrations of legumain were required to induce foam cell formation and related protein expression in THP-1 monocyte-derived macrophages in the present study. This was mostly dependent on the presence or absence of oxLDL. There were also differences in the adequate concentrations of legumain required to elicit different responses among different cells. Cell type-specific signal transduction mechanisms may be responsible for these differences. Further, when the concentration of legumain is higher over the adequate one, it did not exert cytotoxicity. Cells treated with high concentrations of legumain were intact, which were demonstrated by morphological observation, cell variability, and housekeeping gene expression. 

There are some limitations in considering the results from animal experiments. The number of control group was low, because some mice infused with saline died by cannibalism. It was possible that small sample size comparisons did not lead to the statistically significant differences in atherosclerosis and vascular inflammation and remodeling. In addition, the knockout of legumain and administration of legumain inhibitor or neutralizing antibody in *Apoe*^−/−^ mice may strengthen to demonstrate the stimulatory effects of legumain on atherosclerotic vascular remodeling. Likewise, the examination using legumain with its inhibitors or an inactive form of legumain, prolegumain, may be required in all in vitro experiments in future studies. 

The present study gives new insights into the role of legumain in the pathophysiology of atherosclerosis. These results have important clinical implications, identifying legumain as a potential novel target for the treatment of atherosclerosis. Low molecular weight inhibitors and neutralizing antibodies against legumain may become promising candidate drugs for the treatment of atherosclerosis. Several studies have shown that legumain inhibitors suppress cancer metastasis and the pathogenesis of Alzheimer’s disease [14,37,38]. In addition, recent studies have reported that statins (atorvastatin and simvastatin) suppress the expression and activity of legumain in human monocytes/macrophages and in human myotubes [21,39,40]. These interventions against legumain may provide effective therapeutic strategies for preventing atherosclerosis and related vascular remodeling. 

In conclusion, the results from the present study indicated that legumain promoted atherosclerotic vascular remodeling by enhancing macrophage foam cell formation; VSMC migration; and collagen-3, fibronectin, and elastin production by VSMCs. Legumain is expected to emerge as a novel therapeutic target for atherosclerosis and related diseases.

## 4. Materials and Methods

Both in vitro and in vivo experiments were performed in the following manner based on our previous studies [2,5,7,10,30,31,41,42,43,44,45].

### 4.1. Materials

Human recombinant legumain was purchased from Raybiotech (Norcross, GA, USA) for in vitro experiments and from Cusabio (Houston, TX, USA) for in vivo experiments, with the purity of 95% and 90%, respectively. A rabbit polyclonal antibody against human legumain was purchased from Bioss (Woburn, MA, USA). LPS and phorbol 12-myristate 13-acetate were purchased from Sigma (St. Louis, MO, USA) and Wako (Osaka, Japan), respectively. HUVECs and HASMCs were purchased from Lonza (Basel, Switzerland) and THP-1 monocytes were from the Health Science Research Resources Bank (Osaka, Japan).

### 4.2. Reverse Transcription-Polymerase Chain Reaction 

HUVECs were seeded onto 3.5-cm dishes and incubated at 37 °C in 5% CO_2_ for 24 h in EGM-2 [41]. When cells reached ~80% confluence, they were incubated for 30 min with the indicated concentrations of legumain and then for 2 h with 1 μg/mL LPS and 25 ng/mL legumain [2,5,10,42]. *IL6*, *TNFA*, *MCP1*, *ICAM1*, *VCAM1*, *SELE*, and *GAPDH* mRNAs were detected as described previously [2,5,10,30,41,42,43,44]. The sequences of the primers size are listed in Table S1.

### 4.3. Proliferation Assay

HASMCs were seeded onto 96-well plates (1 × 10^4^ cells/100 µL/well) and incubated at 37 °C in 5% CO_2_ for 24 h in SmGM-2 (Lonza). Cells were further incubated for 48 h with the indicated concentrations of legumain, with renewal of each medium. Then, 10 μL of WST-8 solution (Cell Count Reagent SF; Nacalai Tesque, Kyoto, Japan) was added to each well [2,5,7,10,30,31,41,42,43,44,45]. After 1 h of incubation, the amount of formazan product was determined by measuring the absorbance at 450 nm using a Sunrise Remote R™ microplate reader (Tecan, Kawasaki, Japan) [2,5,7,10,30,31,41,42,43,44,45].

### 4.4. Apoptosis Assay 

HASMCs were seeded onto 12-well plates (3 × 10 ^5^ cells/1 mL/well) and incubated at 37 °C in 5% CO_2_ for 24 h in SmGM-2, followed by a 48-h incubation with 25 ng/mL legumain. Cells were then fixed with 4% paraformaldehyde. The TUNEL assay was then performed using an In Situ Apoptosis Detection Kit (Takara Bio, Kusatsu, Japan), as described previously [2,5,10,30,43,44,45].

### 4.5. Migration Assay

HASMCs were seeded onto 8-well culture slides (3 × 10^3^ cells/200 μL/well). Cells were incubated at 37 °C in 5% CO_2_ for 3–5 h in SmGM-2, and were then starved by incubation for 24 h in serum-free SmGM-2. Subsequently, cells were incubated for 15 h in serum-free SmBM, with 25 ng/mL legumain and cells were photographed at 10-min intervals for the last 5 h of the incubation. The average migration distance of 10 cells randomly selected in each well was measured using a BIOREVO BZ-9000 microscope (Keyence, Osaka, Japan) [2,5,7,10,30,31,41,42,43,44,45].

### 4.6. Foam Cell Formation Assay

THP-1 monocytes were seeded onto 3.5-cm dishes (1 × 10 ^6^ cells/1 mL/dish). Cells were incubated at 37 °C in 5% CO_2_ for 3 days in RPMI-1640 medium (Sigma), supplemented with 10% fetal bovine serum, 0.05 mg/mL streptomycin, and 50 U/mL penicillin (monocyte/macrophage conditioning medium [MCM]) and the indicated concentrations of legumain, in the presence of 150 ng/mL phorbol 12-myristate 13-acetate to induce macrophage differentiation [2,5,10,44,45]. THP-1 monocyte-derived macrophages were then incubated for 3 days in MCM with legumain and were further incubated for 2 days in fresh MCM, supplemented with legumain, 50 μg/mL human oxLDL, and 100 μmol/L [^3^H]oleate (PerkinElmer, Yokohama, Japan) conjugated with bovine serum albumin [2,5,7,10,30,31,41,42,43,44,45]. Cellular lipids were extracted and the radioactivity of cholesterol-[^3^H]oleate was determined by thin-layer chromatography [2,5,7,10,30,31,41,42,43,44,45].

### 4.7. Western Blotting 

Aliquots of protein extracts (20 μg) derived from THP-1 monocytes, THP-1 monocyte-derived macrophages, HASMCs, HUVECs, and HUVEC-derived EA.hy926 ECs were separated by 10% sodium dodecyl sulfate-polyacrylamide gel electrophoresis and then immunoblotted with specific antibodies raised against legumain and other proteins, as described previously [2,5,7,10,30,31,41,42,43,44,45]. All antibodies used are listed in Table S2.

### 4.8. Administration of Legumain to Mice

Animal experiments were performed in accordance with the NIH Guidelines for the Care and Use of Laboratory Animals, with protocols approved by the Institutional Animal Care and Use Committee of Tokyo University of Pharmacy and Life Sciences (No. L18-02). In this study, spontaneously hyperlipidemic *Apoe*^−/−^ mice were used as an animal model for atherosclerosis. The *Apoe*^−/−^ mice display poor lipoprotein clearance with subsequent accumulation of cholesterol ester-enriched particles in the blood, which promote the development of atherosclerotic plaques.

A total of 16 male *Apoe*^−/−^ mice (BALB/c. KOR/StmSlc-*Apoe^shl^* mice), at the age of 9 weeks, were purchased from Japan SLC (Hamamatsu, Japan) and maintained on a normal diet until 13 weeks of age, followed by a high-cholesterol diet (Oriental Yeast, Tokyo, Japan) until the experimental endpoint. At 13 and 17 weeks of age, 4 mice with no or minor atherosclerotic lesions in the aorta, respectively, were sacrificed as controls. The remaining 12 mice at 17 weeks of age were divided into 2 groups and these were administered saline (vehicle, *n* = 4) or legumain (5 μg/kg/h, *n* = 8) for 4 weeks using osmotic mini-pumps (Alzet Model 1002; Durect, Cupertino, CA, USA). Once every 2 weeks, the mini-pumps were implanted subcutaneously into the dorsum under medetomidine/midazolam/butorphanol anesthesia [2,5].

At 13, 17, and 21 weeks of age, the *Apoe*^−/−^ mice were sacrificed by exsanguination (total blood collection) under medetomidine/midazolam/butorphanol anesthesia [2,5]. The entire aorta was immediately washed by perfusion with phosphate buffered saline and fixed with 4% formaldehyde. The aorta was excised from the aortic sinus to the abdominal area and connective and adipose tissues were carefully removed [2,5,7,10,30,31,42,43,44,45].

### 4.9. Animal Measurements

Body weight and food intake were measured for *Apoe*^−/−^ mice throughout the study. Systolic and diastolic blood pressures were measured using the indirect tail-cuff method (Kent Scientific, Torrington, CT, USA). At the experimental endpoint, blood samples were collected after a 4-h fast. Plasma concentrations of glucose, total cholesterol, triglycerides, and free fatty acids were measured using commercially available enzymatic methods (Denka Seiken, Tokyo, Japan) [2,5,7,10,30,31,42,43,44,45]. Plasma insulin concentration was measured by enzyme-linked immunosorbent assay (Morinaga, Yokohama, Japan) [2,5,44,45]. 

### 4.10. Assessment of Atherosclerotic Lesions 

The entire aorta luminal surface and cross-sections of the aortic sinus of *Apoe*^−/−^ mice were stained with Oil Red O to assess atherosclerotic lesion area and plaque size, respectively [2,5,7,10,30,31,42,43,44,45]. Atherosclerotic lesions on the aortic tree were expressed as a percentage of the area of lesions relative to the surface area of the entire aorta [2]. Plaque burden was expressed as a percentage of the entire cross-section of the aortic sinus wall [2,30]. In the aortic sinus wall, endogenous legumain expression, vascular inflammation, monocyte/macrophage infiltration, VSMCs, collagen-3 expression, and collagen fibers (total collagen) were visualized by staining with antibodies for legumain, pentraxin-3 (Bioss), MOMA-2 (Millipore, Billerica, MA, USA), α-SMA (Sigma), or collagen-3 (GeneTex, Irvine, CA, USA) or Masson’s Trichrome (Muto Pure Chemicals, Tokyo, Japan), respectively [2,5,7,10,30,31,42,43,44,45]. The macrophage (μm^2^)/VSMC (μm^2^) ratio within atheromatous plaques was calculated to evaluate plaque stability [5,30,43,44,45]. The positively stained areas were traced by an investigator, blind to the treatment, and were quantified by image analysis using Photoshop (Adobe, San Jose, CA, USA) and ImageJ (NIH, Bethesda, MD, USA). 

### 4.11. Assessment of Legumain Expression in Intimal Lesions following Wire Injury

Under deep anesthesia, vascular injury was induced with a spring guide wire (diameter: 0.014 inches) in the left femoral artery of 12-week-old *Apoe*^−/−^ mice fed a high-cholesterol diet [10]. Three weeks after the procedure, the mice were sacrificed to determine arterial neointimal thickness [10]. Paraffin-embedded cross-sections of wire-injured and non-injured femoral arteries were stained with Elastica-Van Gieson and an anti-legumain antibody.

### 4.12. Statistical Analysis

All values are expressed as means ± SEM. Data were analyzed by unpaired Student’s *t*-test for 2 groups and by one-way analysis of variance, followed by Bonferroni’s post hoc test, for ≥3 groups, using Statview-J 5.0 (SAS Institute, Cary, NC, USA). Statistical significance was defined as *p* < 0.05.

## Figures and Tables

**Table 1:** Characteristics and laboratory data of *Apoe*^−/−^ mice.

	Saline (*n* = 4)	Legumain (*n* = 8)	*p*-Value
Body weight (g)	29.8 ± 0.8	27.9 ± 0.8	0.1818
Food intake (g/d)	3.05 ± 0.20	3.05 ± 0.23	0.9854
Systolic blood pressure (mm Hg)	97.0 ± 3.2	99.5 ± 2.6	0.5750
Diastolic blood pressure (mm Hg)	72.8 ± 3.5	75.4 ± 2.6	0.5664
Glucose (mg/dL)	260.6 ± 35.2	216.2 ± 14.8	0.1942
Total cholesterol (mg/dL)	1342.1 ± 41.4	1607.7 ± 123.4	0.1729
Triglycerides (mg/dL)	242.9 ± 34.6	209.9 ± 19.7	0.3907
Free fatty acids (mEq/L)	2.08 ± 0.25	2.18 ± 0.21	0.7825
Insulin (ng/mL)	0.21 ± 0.05	0.09 ± 0.04	0.0966

Data are expressed as mean ± SEM. Analyzed by unpaired Student’s *t* test.

## Supplementary Materials

Supplementary materials can be found at https://www.mdpi.com/1422-0067/20/9/2195/s1.

## Abbreviations

ABCA1	ATP-binding cassette transporter A1
ACAT-1	acyl-coenzyme A:cholesterol acyltransferase-1
*Apoe*^−/−^ mice	apolipoprotein E-deficient mice
EC	endothelial cell
ECM	extracellular matrix
HASMC	human aortic smooth muscle cell
HUVEC	human umbilical vein endothelial cell
ICAM-1	intercellular adhesion molecule-1
IL	interleukin
LPS	lipopolysaccharide
MCM	monocyte/macrophage-conditioned medium
MCP-1	monocyte chemoattractant protein-1
MMP	matrix metalloproteinase
NCEH	neutral cholesterol ester hydrolase
NF-κB	nuclear factor-κB
OxLDL	oxidized low-density lipoprotein
SR-A	scavenger receptor class A
TNF-α	tumor necrosis factor-α
VCAM-1	vascular cell adhesion molecule-1
VSMC	vascular smooth muscle cell
