# Supplementary material for: Legumain Promotes Atherosclerotic Vascular Remodeling
Source: Int J Mol Sci. 2019 May 4;20(9):2195. doi: 10.3390/ijms20092195 (PMC6539540; doi:10.3390/ijms20092195)
Supplement: Supplementary file 1 [file ijms-20-02195-s001.pdf]

Supplementary Table 1. Primer sequences used for reverse transcription-polymerase chain reaction.

Gene		Primer Sequence (5’→3’)	Product Size (bp)
IL6	Forward	ATGAACTCCTTCTCCACAAGCGC	628
	Reverse	GAAGAGCCCTCAGGCTGGACT	
TNFA	Forward	CAGAGGGAAGAGTTCCCCAG	325
	Reverse	CCTTGGTCTGGTAGGAGACG	
MCP1	Forward	CAATAGGAAGATCTCAGTGC	189
	Reverse	GTGTTCAAGTCTTCGGAGTT	
ICAM1	Forward	CGACTGGACGACAGGGATTGT	290
	Reverse	ATTATGACTGCGGCTGCTACC	
VCAM1	Forward	TCCCTACCATTGAAGATACTGGAAA	146
	Reverse	GCTGACCAAGACGGTTGTATCTC	
SELE	Forward	CCTACAAGTCCTCTTGTGCCTTC	206
	Reverse	ACAGGCGAACTTGCACACA	
GAPDH	Forward	ACCACAGTCCATGCCATCAC	451
	Reverse	TCCACCACCCTGTTGCTGTA	

IL6 = interleukin-6, *TNFA* = tumor necrosis factor-α, *MCP1* = monocyte chemoattractant protein-1, *ICAM1* = intercellular adhesion molecule-1, *VCAM1* = vascular cell adhesion molecule-1, *SELE* = endothelial selectin, *GAPDH* = glyceraldehyde-3-phosphate dehydrogenase.

Supplementary Table 2. Antibodies used for Western blotting.

Protein	Manufacturer	Cat. #	Dilution
Legumain	Bioss	bs-3907R	1:500
GAPDH	Acris	ACR001PS	1:5000
CD68	Santa Cruz Biotechnology	sc-17832	1:250
MARCO	Bioss	bs-2659R	1:500
Arginase-1	GeneTex	GTX109242	1:1000
β-Actin	Sigma	A1978	1:2000
CD36	R&D Systems	AF1955	1:1000
SR-A	R&D Systems	MAB2708	1:500
ACAT-1	Santa Cruz Biotechnology	sc-20951	1:2000
NCEH	ProteinTech	14021-AP	1:1000
ABCA1	Novus Biologicals	NB400-105	1:500
Collagen-1	Novus Biologicals	NB600-408	1:1000
Collagen-3	GeneTex	GTX102997	1:1000
Fibronectin	GeneTex	GTX112794	1:1000
Elastin	Bioss	bs-1756R	1:500
MMP-2	GeneTex	GTX104577	1:1000
MMP-9	EnoGene	E11-0275C	1:500
PI3K	Abcam	Ab40755	1:1000
p-Akt	Cell Signaling Technology	9614	1:1000
p-ERK1/2	Cell Signaling Technology	4376	1:1000
p-JNK	GeneTex	GTX52327	1:500
p-p-38	Cell Signaling Technology	4511	1:1000
p-NF-κB	GeneTex	GTX50254	1:500
α-Tubulin	GeneTex	GTX112141	1:1000

GAPDH = glyceraldehyde-3-phosphate dehydrogenase, SR-A = scavenger receptor class A, ACAT-1 = acyl-coenzyme A:cholesterol acyltransferase-1, NCEH = neutral cholesterol ester hydrolase, ABCA1 = ATP-binding cassette transporter A1, MMP = matrix metalloproteinase, PI3K = phosphoinositide 3-kinase, ERK = extracellular signal-regulated kinase, JNK = c-jun N-terminal kinase, NF-κB = nuclear factor-κB.
